# Supplementary material for: Factors influencing gestational diabetes self-care among pregnant women in a Syrian Refugee Camp in Jordan
Source: PLoS One. 2024 Feb 15;19(2):e0297051. doi: 10.1371/journal.pone.0297051 (PMC10868830; doi:10.1371/journal.pone.0297051)
Supplement: Attachment — Submitted filename: GDM-main article PLOS ONE.docx [file pone.0297051.s002.docx]

**Factors Influencing Gestational Diabetes Self-Care Among Pregnant Women in a Syrian Refugee Camp in Jordan**

Enas A. Assaf^1*^, Haleama Al Sabbah^2^, Aaliyah Momani^1^, Rasmiyeh Al-Amer^3^, Ghada A Al-Sa’ad^4^, Anas Ababneh^1^

^1^ Faculty of Nursing, Applied Science Private University, Amman, Jordan

^2^College of Health Sciences, Abu Dhabi University, United Arab Emirates

^3^ School of Nursing, Al-Israá University, Amman, Jordan

^4^Jordan Health Aid Society/UNFPA, Za’atari Camp, Jordan

*Corresponding author

Enas A. Assaf

Faculty of Nursing, Applied Science Private University ,A l Arab st. 21-Amman -Jordan

Tel : 00962-65609999, Post Code : 11937, P.O.Box 541350

e-mail: [e_assaf@asu.edu,jo](mailto:e_assaf@asu.edu,jo)

**Abstract**

**Aim**

The main objective of this study is to identify the level of self-care practices and the determinants of GDM among pregnant women residing in one of the refugee camps in Jordan.

**Method**

A cross-sectional study was conducted on a convenient sample of forty women diagnosed with GDM from the high-risk maternity clinic in one of the Syrian refugee camps in Jordan. The study used the Gestational Diabetes Management Self-Efficacy Scale (GDMSES), Diabetes Knowledge (DMK) assessment, and Diabetes Self-Care Activities Questionnaire (SDSCA) to measure the variables of interest.

**Results**

Significant associations were found between the subcategories of diet, exercise, and blood control in both the self-efficacy and self-activity scales (p < 0.01, p < 0.01, p < 0.5), respectively. Two factors were associated with higher GDM self-care: diabetes knowledge and higher self-efficacy toward GDM self-care (p < 0.5).

**Conclusion**

The findings of this study highlight that pregnant women with GDM who have higher levels of self-efficacy and diabetes knowledge are more likely to achieve higher levels of GDM self-care. Beside developing health promotion programs to enhance women's self-efficacy in adhering to GDM care, adequate support and relevant resources to facilitate GDM management among refugee women are recommended.

**Keywords**: Gestational Diabetes Mellitus; Self-care; Self-efficacy; Pregnancy; Refugee Camp

**Introduction**

Gestational Diabetes Mellitus (GDM) is glucose intolerance that usually begins or is diagnosed first during pregnancy, and in most cases, it ceases post-delivery [1]. In the United States, it affects yearly around 2-10% of women [2, 3]. In Europe, it was reported to affect 5.4% of women [4]. Furthermore, a high prevalence of GDM (~13%) was found in Middle Eastern countries and North African countries [5]. In Jordan, one study conducted at the Jordan University Hospital from January 2015 to January 2016 showed a high prevalence of (13.5%) of GDM [6].

GDM is found to be associated with an increased risk of adverse fetal outcomes such as neonatal hypoglycemia and macrosomia, as well as maternal complications, including; gestational hypertension, premature rupture of membranes, vaginal candidiasis, and preterm birth [7, 8]. Additionally, women diagnosed with GDM have an increased likelihood of developing type 2 diabetes within 5-10 years post-delivery [9]. Women diagnosed with GDM typically experience a progression through several stages in dealing with the condition, including shock upon receiving the diagnosis, coming to terms with GDM, acquiring new strategies to manage GDM, future planning, and seeking environmental support [10].

Managing GDM can be particularly challenging as it may rely on women's adherence to care [11]. Therefore, it is essential to consider the cultural factors that affect self-care behaviors among women with GDM [12]. The management of GDM includes physical activity; nutritional monitoring and control; glucose monitoring; and pharmacological therapy [13]. Poor self-care behaviors of GDM might increase the risk for comorbidities for both the infant and the mother [10]. Self-care behaviors among women with GDM can be influenced by; multiple socio-demographic, physiological, and psychosocial factors. These factors could facilitate the management, such as; mothers’ concerns about their newborn’s health and social support, whereas some factors could be barriers to management, for example, physical and social constraints and limited comprehension of GDM management requirements [14, 15]. Based on the social cognitive theory (SCT) [16] that predicts health behaviors by adopting personal cognitive factors such as socio-structural factors (facilitators or impediments), self-efficacy (a person’s expectations of own ability to perform behaviors), outcomes expectations (person’s belief of the outcomes that result from performing behaviors) [17], which overall could predict women’s self-care behaviors.

Jordan is the sixth largest refugee host country in the world [18]. To escape military conflict in Syria since 2011, millions of Syrians fled to other countries, including Jordan. Jordan hosts over 673 thousand Syrian refugees, 19.5% of whom reside in the United Nations Refugee Agency’s three camps; the Za’atari refugee camp is the largest one [19].

Syrian refugees residing in camps in Jordan have lower perceived health compared to other refugees living outside [20]. Healthcare services in these camps are provided by multiple charitable organizations and are offered free of charge [21]. However, some refugees lack access to healthcare services due to structural barriers such as lacking appropriate civil documents [22, 23]. These services focus on communicable and non-communicable diseases, mental health, and women’s health, including reproductive health services and the associated issues with child marriages and sexual violence [24]. However, women’s health needs and services among Syrian refugees require further attention and improvement [25]. To the best of our knowledge, no studies published to date on GDM among Syrian refugees. Self-management among Syrian refugees was found among patients with type 2 diabetes in Bekaa Valley in Lebanon to find out that knowledge and education related to diabetes, secondary schooling education level, higher patient self-confidence in self-care as well as insulin use were highly associated with higher scores of diabetes self-management, whereas, a lower score of self-management found to be associated with increasing age and with those who diagnosed during the Syrian conflict [26].

In Turkey, it has been observed that Syrian refugees have lower rates of gestational diabetes testing and antenatal visits. Additionally, Syrian refugee women experience more complicated pregnancies with higher rates of preterm births, rupture of membranes, and low birth weight [27]. Studies conducted in Jordan and Turkey have also shown that Syrian refugee women have significantly higher rates of preterm birth and low birth weight compared to Jordanian and Turkish women, respectively [28, 29]. Furthermore, Boulle and colleagues [30] have identified significant challenges in providing diabetic care by humanitarian organizations in crisis situations, including limited access to essential medicines and diagnostic resources necessary for clinical care. It is crucial to enhance the level of care provided in these circumstances.

In Jordan, several studies investigated the self-care/ self-management of people with diabetes [31-35]. Al- Khawaldeh et al. [32] found that participants with higher self-efficacy showed better self-management behaviors in diet, blood sugar testing, exercise, and medication. However, there is a scarcity of studies concerning Syrian women and pregnant women with GDM residing in the refugee camps which would help policymakers and donner organizations in prioritizing the health care delivery plans in improving the pregnancy outcomes among this displaced group. Therefore, this study aims to measure the levels of self-care activities of Syrian women with GDM residing in the Za’atari refugee camp and its associated factors.

**Methods**

***Study Design and sampling and setting***

A cross-sectional study was conducted on a convenience sample of 40 pregnant women residing in the Za'atari refugee camp in Jordan between September and December 2022. The Za'atari camp, established in 2012, is the largest Syrian refugee camp in Jordan, located approximately 10 kilometers east of Mafraq city in the northern region. The camp hosts over 80,000 residents [19]. By comparing Jordanian and Syrian women in terms of maternal health care, the study revealed that, on average, Syrian women received fewer antenatal care visits when compared to Jordanian women (62% and 82%, respectively) [36].

According to sample size calculation guidelines, it is commonly recommended that for regression analysis, there should be a minimum of 10 observations per variable. As we are utilizing three main variables for regression analysis, a minimum sample size of 30 is required [37]. The study included all pregnant women diagnosed with gestational diabetes mellitus (GDM) at a gestational age of more than 20 weeks. However, women with other pregnancy-related comorbidities or pre-existing chronic conditions like Type 1 or Type 2 diabetes mellitus were excluded. Additionally, women who were illiterate or had been diagnosed with mental illness were also excluded from the study.

***Ethical consideration***

Prior to administering the questionnaire, a consent form was obtained from each participant, ensuring they were adequately informed about the study. The researcher was available to address any questions or concerns during the process. The study obtained approval from the Scientific Research Ethical Committee of the Faculty of Nursing at Applied Science Private University (IRB no. Faculty 2021-2022-44) and also received ethical approval from the Ministry of Interior Affairs (no. 5/MT/19166/49608). Furthermore, necessary approvals were obtained from the camp security and clinic management service to conduct the study.

***Data collection and study setting***

The study was conducted at the reproductive health clinic-Z3, managed by the Jordan Health and Society (JHASI) at the Za’atari camp in Jordan. This clinic provides comprehensive care for all high-risk pregnant women in the camp, including those diagnosed with GDM. On average, the clinic receives approximately 200 high-risk pregnant women monthly (around 10% of them being diagnosed with GDM). Data collection was carried out by a trained research assistant who was available to address any inquiries or concerns from the women, considering that these women had only completed primary school education, reflecting the educational background within the camp. Women who met the inclusion criteria were approached by the research assistant at the registration desk in the clinic. The research assistant explained the research objectives and ensured the strict confidentiality of personal information. Permission to participate in this study was obtained from all women. They believe this study would significantly improve their health status and the healthcare services provided to them.

*Study tool*

A self-administered questionnaire was utilized in the study, comprising seven sections: *Section One:* Sociodemographic Characteristics - This section captured information regarding age, education, occupation, and socio-economic status. *Section Two*: General Health History and Family History of Diabetes - Participants were asked to provide details about their overall health history and any family history of diabetes. *Section Three*: Pregnancy Information - This section was focused on the current pregnancy, including the Last Menstrual Period (LMP), gestational age, gravidity, parity, activities of daily living (ADLs), and timing and location of antenatal visits. *Section Four:* GDM Profile and Pre-pregnancy BMI - Participants were requested to provide their GDM profile and self-reported BMI before pregnancy, including height in centimeters and weight in kilograms. BMI was calculated using the World Health Organization (WHO) formula and recommended specifications (underweight < 18.5, normal weight 18.5-24.9, pre obesity 25-29.9, obesity >30) [38]. Additionally, it included fasting blood sugar, glucose tolerance test results, gestational age at GDM diagnosis, HbA1C levels which is categorized into two categories (poor self-care >6.5, and good self-care ≤6.5) [39], and blood pressure. *Section Five:* Diabetes Knowledge Questionnaire (DKQ) in Arabic [40] - The DKQ consisted of 24 items with multiple-choice questions. Participants received one point for each correct answer and zero for incorrect responses. The total score ranged from 0 to 24, with higher scores indicating a higher level of diabetes knowledge. The DKQ assessed various aspects, including general knowledge about diabetes (nine items), physical activity (two items), medications (four items), nutrition (three items), complications (two items), foot care (two items), sick day management (one item), and follow-up and blood monitoring (one item). The DKQ demonstrated good content validity with a Content Validity Index (CVI) of 0.92 and a Cronbach's alpha coefficient of 0.78. *Section Six*: Health Education on GDM - Participants reported whether they received health education on GDM, including topics such as definition, diet management, exercise management, pharmacological care, self-glucose monitoring, and sources of health education. *Section Seven*: Gestational Diabetes Management Self-Efficacy Scale (GDMSES) [41] .This scale, translated into Arabic, consisted of 16 Likert scale items rated from 0 to 10. It measured women's confidence in performing various activities related to GDM management. The scale's score interpretation was as follows: 0-3: low confidence, 4-8: moderate confidence, 9-10: high confidence. The scale exhibited good internal consistency reliability (Cronbach's alpha = 0.85) and stability over time (Pearson correlation coefficient > .6). Section Eight: Diabetes Self-Care Activities (SDSCA) [42] - This instrument assessed the diabetes self-care activities of individuals with diabetes. The Arabic translation of the scale demonstrated acceptable reliability (α= 0.68) [43]. The scale evaluated the frequency of performing different self-care activities over the preceding week. Response options ranged from 0 to 7, with higher scores indicating better adherence to self-care activities. The scale comprised 14 items covering seven constructs of diabetes self-care activities: physical activity (2 items), general and specific diet (4 items), blood sugar measurement (2 items), medication (3 items), foot care (2 items), and adherence and smoking behaviors (1 item).

**Statistical analysis**

*Explanatory variables:* The study examined various variables as potential explanatory factors, including sociodemographic characteristics, health-related variables (health history and obstetrical health, GDM-related health variables), and psychosocial variables. Sociodemographic variables: Participants self-reported information on age, marital status, family income, employment, education level, living place, and smoking status. Health-related variables: GDM-related health variables included the type and duration of diabetes, self-reported body mass index (BMI) before pregnancy, HbA1c, glucose tolerance test, gestational age at the time of diagnosis, and details of diabetes treatment. Psychosocial factors examined in the study included participants' knowledge about GDM, self-efficacy in managing GDM, and self-care practices.

*Statistical analysis:* The data were analyzed using IBM SPSS Statistics version 23.0 for Windows (IBM Corp, Armonk, NY, USA). This included descriptive analysis such as frequencies (proportions), mean (standard deviation (SD)), and median (interquartile range (IQR)). Pearson correlation analysis was used to assess the associations between the subclasses of self-efficacy and the subclasses of self-care. Multiple linear regression analysis was used to examine the association of the subset of sociodemographic variables, health variables, self-efficacy score, and knowledge score (predictors) with the self-care score (outcome). Statistical significance was considered when the p-value was less than 0.05.

**Results**

A total of 40 pregnant women participated in the study survey. Table 1 presents the sociodemographic and health characteristics of the participants. The mean age of the women is 33.2 years. The majority of them are unemployed and have completed primary school education. Additionally, they were non-smokers. About 60% of the participants had previously experienced GDM, with an average of 6.7 pregnancies. The majority of women (37 women ) had their last checked HbA1C was higher than 6.5mmol/L which indicated poor GDM management. The mean reported pre-pregnancy BMI is 25.2. and almost all women were within normal BMI ranges. Regarding their knowledge, the average number of correct answers to the knowledge questions was 10. In addition, more than half of the women (57%) reported receiving health education from the clinic, specifically about GDM. On the one hand, the highest self-efficacy sub-score was related to exercise (mean: 3.91 ± 2.96), indicating a higher level of confidence in this area. On the other hand, the lowest mean score was observed in the diet category (mean: 3.16 ± 2.64) (Table 1).

Table 1. Characteristics of the study participants (number (%) or mean ±SD) unless otherwise stated*

| **Characteristics** | **Total (n=40)** |
| --- | --- |
| **Sociodemographic characteristics** |  |
| Age (years) | 33.2 ±6.5 |
| Primary school education | 36 (90%) |
| Unemployed | 38 (95%) |
| Number of children* | 5 (3-6) |
| **Health characteristics and activities** |  |
| Hypertension | 8 (20%) |
| Thyroid disease | 4 (10%) |
| Previous history of GDM | 24 (60%) |
| Gestational age | 38 (34-39)* |
| Gravida | 6.7 ± 2.7 |
| Para | 4.9 ±2.4 |
| **BMI (before pregnancy)**  Underweight : <18.5  Normal weight:: 18.5-24.9  Pe-obesity: 25-29.9  Obesity >30 | **25.2± 3.5**  0 (0.0%)  19 (47.5%)  18 (45%)  3 (7.5%) |
| **HbA1C (%, mmol/L)**  Poor self-care (>6.5mmol/L)  Good self-care (<6.5mmol/L) | **7.3 ±1.6**  37(92.5%)  2(5%) |
| Regular antenatal following | 36 (90%) |
| **Knowledge on diabetes? Or general health** |  |
| Prior heath education | 23 (57.5%) |
| Knowledge about diabetes | 10 ±5.6 |
| **Self-efficacy (GDMSES)** |  |
| Score of Self-efficacy to GD self-care (GDMSES)  Self-efficacy subclasses  Diet  Exercise  Blood control | **1.39 ±1.09**  3.16±2.64  3.91±2.96  3.54 ±2.48 |
| **Score of (SDSCA)** | **1.39 ±1.09** |

* *Median (min-max)*

***Knowledge questionnaire (KDM)*** ***Diabetes Knowledge Questionnaire (DKQ)?***

The mean score for the correctly answered questions in the KDM questionnaire was 10 ± 5.6. The highest percentage of correct answers was observed in answering the question related to the best method of measuring blood sugar levels (75%), followed by the question regarding the effect of exercise on blood sugar levels, with a correct response rate of 62%. Conversely, the lowest percentage of correct answers was provided for the question related to the effects of consuming sugary juices on diabetes mellitus (12.5%) (Table 2).

Table 2. Percentages and Frequences of total correct answers in the knowledge questionnaire (KDM)

| Knowledge scale questions  *(Multiple choice 24 questions)* | *N(40)* |
| --- | --- |
|  | correct  n (%) |
| What is HBA1C | 20 (50.0%) |
| What is the normal level of sugar in blood | 18 (45.0%) |
| Best method to measure level of sugar | 30 (75.0%) |
| What is DM | 11 (27.5%) |
| Which of the following is a correct statement regarding DM | 13 (32.5%) |
| Choose the signs of high blood sugar | 18 (45.0%) |
| Choose signs of low blood sugar | 7 (17.5%) |
| Choose reasons for low blood sugar | 10 (25.0%) |
| Choose the actions that you should do in case of low blood sugar | 20 (50.0%) |
| DM tablet medication effect is | 18 (45.0%) |
| Best sites to take insulin injection are | 17 (42.5%) |
| Where you should keep insulin vial of pen after use | 19 (47.5%) |
| Choose the best statement describe DM medication | 16 (40.0%) |
| What you should do when you feel can’t eat sufficient food | 14 (35.0%) |
| What is the effect of exercise on the level of blood sugar | 25 (62.5%) |
| DM patients are advised to perform exercises | 15 (37.5%) |
| What is the effect of drinking juices high in sugar | 5 (12.5%) |
| Which of the following items never use for treating low blood sugar | 22 (55.0%) |
| Choose the best statement about diet restrictions for DM | 15 (37.5%) |
| DM patients are advised to check their blood sugar regularly because | 18 (45.0%) |
| Which of these problems are not related to DM | 21 (52.5%) |
| Choose the complications of DM | 16 (40.0%) |
| The reasons why foot complications are noticed among DM patients | 21 (52.5%) |
| Best way to make foot care | 11 (27.5%) |
| Total Knowledge score (mean ±SD) | **10 ±5.4** |

***Gestaional Diabete Self-Care (SDSCA)***

Table 3 presents the mean scores for each item in the GDSS (Gestational Diabetes Self-Management Scale). The highest-scoring activities reported by women in the last 7 days were consuming a high-fat diet and eating fruits and vegetables. Whereas, the activities with the lowest scores were blood sugar testing and engaging in specific exercise sessions.

Table 3. Score of self-care activities of the study participants in the last 7 days (n=40) (Mean±SD)

| **Items** | **Mean (SD)** |
| --- | --- |
| **General diet**  Item 1: Follow healthغ diet. | **2.43 (1.83)**  1.7 (2.56) |
| Item 2: Follow own diet. | 1.87 (2.56) |
| Item 3: Eats high fat diet. | 3.9 (3.07) |
| Item 4: Eats fruits and vegetables | 2.25 (2.40) |
| **Exercise**  Item 5: Participates in at least 30 minutes of physical activity per day | **1.5 (1.65)**  2.4(2.75) |
| Item 6: Participates in specific exercise sessions | 0.6 (1.5) |
| **Blood sugar testing**  Item 7: Tests for blood sugar | **0.57 (1.23)**  0.65 (1.33) |
| Item 8: Tests for blood sugar as recommended by healthcare provider | 0.5 (1.2) |
| **Foot care**  Item 9: Checks feet | **0.92 (1.96)**  1.1 (2.24) |
| Item 10: Inspects the inside of shoes | 0.75(1.93) |
| **Adherence to treatment**  Item 11: Takes diabetes medication | **1.2 (1.55**)  1.43(2.62) |
| Item 12: Takes insulin | 0.95 (2.33) |
| Item 13: Takes diabetes pills | 1.45 (2.56) |
| **Smoking**  Item 14: Smokes | 0.3 (0.16) |

***Self-efficacy and self-care subclasses correlations***

Table 4 presents the correlation coefficients between the subclasses of the self-efficacy and

the subcategories of self-care were examined, revealing significant associations. A strong positive correlation was found between self-efficacy in diet and adherence to dietary self-care (r = .64, p < 0.01). Similarly, a strong positive correlation was found between self-efficacy in exercise and engagement in exercise-related self-care (r = .67, p < 0.01). Furthermore, a significant positive correlation was identified between self-efficacy in blood control and adherence to blood control self-care (r = .35, p < 0.05).

**Table 4.** Correlation coefficients between self-efficacy subclasses and corresponding self-care subclasses

|  |  | Self-efficacy subclasses | | |
| --- | --- | --- | --- | --- |
|  |  |  | | |
|  |  | Diet | Blood control | exercise |
| Self-care subclasses | Diet | .637** | .71** | .560** |
|  | Blood control | .295ns | .353* | .279ns |
|  | exercise | .641** | .62** | .669** |

ns: not significant, *: p < 0.5, **: p < 0.01

***Self-care predictors***

Table 5 shows the results of multiple regression analysis to test if sociodemographic and health variables, self-efficacy score, and knowledge score significantly predicted self-care scores. The overall regression model was significant (R2 = 0.56, p = 0.006). It was found that the self-efficacy score to SDSCA significantly predicted self-care score (β =0.458, p = 0.016). It was also found that knowledge about diabetes significantly predicted self-care scores (β = 0.336, p = 0.031). However, sociodemographic and health variables did not significantly predict the self-care score.

Table 5. Multiple linear regression of sociodemographic selected variables, health variables, self-efficacy score, and knowledge score on self-care.

| **Independent variables** | **Standardized coefficients** | **significance** |
| --- | --- | --- |
| Age | .103 | .556^ns^ |
| Education | -.173 | .606^ns^ |
| Occupation | .156 | .623^ns^ |
| History of GD | -.235 | .159^ns^ |
| Gravida | -.044 | .901 ^ns^ |
| Para | -.085 | .928 ^ns^ |
| No. of children | -.065 | .944 ^ns^ |
| HbA1C | -.066 | .650 ^ns^ |
| BMI before pregnancy | .190 | .190 ^ns^ |
| Self-efficacy to GDSC | .458 | .016^*^ |
| Knowledge about diabetes | .336 | .031^*^ |
|  |  |  |
| **Model summary** |  |  |
| R^2^ | = .559 |  |
| F-ratio | = 3.23 |  |
| p-value | P < .006 |  |

ns: not significant, *: p < 0.5

**Discussion**

The main objective of this study was to assess the levels of self-care and their associations among refugee women with GDM in Za’atari camp, Jordan. The mean score for self-care among the women was 1.39 ± 1.09, while the mean score for self-efficacy was also 1.39 ± 1.09. Two factors were identified to be associated with GDM self-care, which are: self-efficacy and knowledge. Social Cognitive Theory [16] suggests predicting the health behavior of individuals based on two factors: self-efficacy (an individual’s belief of their own ability to perform the behavior) and outcomes expectations (an individual’s belief of the outcomes that result from performing a health behavior) [17].

The self-care score assesses the activities performed by pregnant women in the past 7 days, with higher scores indicating better adherence to self-care practices. Among the self-care subclasses, the highest scores were observed in the diet category, followed by adherence to treatment, while the lowest score was found in blood control. These sub- scores were lower compared to the baseline data from an intervention study conducted in Oman, where the highest score was reported for diet (4 ± 1.3), and the lowest score was observed for physical activity (2.5 ± 2.0) [44].

Among the various subscales related to diet adherence, the lowest level of adherence was found in following the prescribed diet plan compared to other subitems. This can be attributed to the lack of health education and support, and limited access to dietary resources especially for refugee women. These women are typically required to follow the same diet as their families, which is primarily supported by the UNHCR food distribution system. A quasi-experimental study conducted among diabetic patients who were Syrian refugees in Jordan demonstrated that a combination of cash assistance and health education was essential for improving effective care and diabetic control. The provision of cash assistance would enable better diet control and facilitate more frequent visits and follow-ups [45].

Blood glucose control was the least performed subscale in terms of self-care, the main reason for that result is that in the clinic, women receive no health education in that regard since GDM women do not receive strips used for self-monitoring for blood glucose level, and women do not check their blood glucose levels regularly at home, the only chance for them to check their blood sugar is during their antenatal visits, which might take place for high-risk pregnancy women every two to three weeks; however, only patients with DM type 1 or 2 receive strips from the clinic for follow-up and control [45].

Interestingly, the highest score on the knowledge scale was among the question concerning the best method to measure blood glucose (75%) which supports the idea that low self-care activity was related to a lack of resources and affordability. One study among Syrian refugees in Turkey found that only 15% of the participants conducted GDM screening tests since that was costly, not all women afford it, and not covered by humanitarian organizations [27]. Our study found two predictors for GD self-care; self-efficacy and knowledge. Self-efficacy sub-scales for diet, exercise, and blood glucose control were highly significant, with similar corresponding subscales. This was similar to a study conducted among DM patients with type 2 self-management for glycemic control in Jordan[46].

Concerning GDM, it was found among other studies worldwide a strong positive relationship between self-efficacy and GDM self-control [47-51]. Self-efficacy was also highly correlated with improving self-management among individuals with DM in several studies [52-57]. Hence, enhancing women's self-efficacy regarding diet control, exercise, and blood glucose control holds the potential to improve their self-management. Patients with high self-efficacy are more inclined to establish goals that facilitate behavioural changes and long-term adherence to new behaviors. This, in turn, may contribute to better overall health outcomes [58]. Based on the social learning theory and cognitive theory, health promotion is greatly influenced by an individual's attitudes and beliefs regarding their ability to perform specific activities correctly. Therefore, the adherence to and maintenance of new health behaviors is more likely to be influenced by stronger self-efficacy [59].

Knowledge was another significant predictor found to be associated with self-care among our studied women, knowledge and educational programs were strongly correlated with self-efficacy in other chronic diseases [60]. Therefore, knowledge might be a core effect affecting women’s self-efficacy and improving their self-confidence. A study carried out by Saboula and her colleagues [61] in Egypt found that the level of poor knowledge of GDM was 90% among pregnant women, while after an intervention study, it changed to 10%; the study recommended continuous health education for GDM as better knowledge increased self-care activity.

***Strengths and Limitations***

The novelty of this particular study lies in the fact that Syrian refugee pregnant women with GDM have not been thoroughly researched. The significant factors pertaining to GDM self-care were shown in this study. Although future research could take note of the relatively small sample size, this study's strength in originality persists.***Conclusion and Recommendations***

This study showed that women who perceived higher self-efficacy are a potential predictor of better GDM self-care. Refugee women need more attention and support from humanitarian organizations, clinicians, and policymakers to improve women’s knowledge and develop new special programs to enhance women’s self-efficacy in adherence to GDM care and management, as well as support relevant resources to improve maternal and neonatal outcomes. Furthermore, there is a need for future research to explore refugee women’s experience of GDM.

**Acknowledgements**

We extend our gratitude to the dedicated research assistant who played a crucial role in promptly collecting the data. Special thanks go to all the study participants for their valuable participation and time devoted to completing the questionnaire. Our sincere appreciation and acknowledgment go to the reproductive health clinic-Z3 and its management body, as well as the Jordan Health and Society (JHASI) at Al Za’atari Camp for their cooperation throughout the study. Furthermore, we would like to acknowledge the facilitation provided by the security personnel at the Al Za’atari camp, which contributed to the smooth progress of the research process.

**Data availability**: All data are fully available without restrictions

**References**

1.	Ali N, Aldhaheri AS, Alneyadi HH, Alazeezi MH, Al Dhaheri SS, Loney T, et al. Effect of Gestational Diabetes Mellitus History on Future Pregnancy Behaviors: The Mutaba'ah Study. International journal of environmental research and public health. 2020;18(1). Epub 2020/12/31. doi: 10.3390/ijerph18010058. PubMed PMID: 33374764; PubMed Central PMCID: PMCPMC7796159.

2.	Spaight C, Gross J, Horsch A, Puder JJ. Gestational Diabetes Mellitus. Endocrine development. 2016;31:163-78. Epub 2016/01/30. doi: 10.1159/000439413. PubMed PMID: 26824237.

3.	ACOG Practice Bulletin No. 190: Gestational Diabetes Mellitus. Obstetrics and gynecology. 2018;131(2):e49-e64. Epub 2018/01/26. doi: 10.1097/aog.0000000000002501. PubMed PMID: 29370047.

4.	Eades CE, Cameron DM, Evans JMM. Prevalence of gestational diabetes mellitus in Europe: A meta-analysis. Diabetes Research and Clinical Practice. 2017;129:173-81. doi: <https://doi.org/10.1016/j.diabres.2017.03.030>.

5.	Zhu Y, Zhang C. Prevalence of Gestational Diabetes and Risk of Progression to Type 2 Diabetes: a Global Perspective. Current diabetes reports. 2016;16(1):7. Epub 2016/01/09. doi: 10.1007/s11892-015-0699-x. PubMed PMID: 26742932; PubMed Central PMCID: PMCPMC6675405.

6.	Basha AS, Fram KM, Thekrallah FM, Irshaid ZA, Maswady AaM, Obeidat ZNJIJoDiDC. Prevalence of gestational diabetes and contributing factors among pregnant Jordanian women attending Jordan University hospital. 2019;39(1):132-8.

7.	Zhuang W, Lv, J., Liang, Q., Chen, W., Zhang, S., & Sun, X. Adverse effects of gestational diabetes-related risk factors on pregnancy outcomes and intervention measures. Exp Ther Med Experimental and therapeutic medicine. 2020;20(4):3361-7.

8.	Kouhkan A, Najafi, L., Malek, M., Baradaran, H. R., Hosseini, R., Khajavi, A., & Khamseh, M. E. Gestational diabetes mellitus: Major risk factors and pregnancy-related outcomes: A cohort study. Int J Reprod Biomed International journal of reproductive biomedicine. 2021;19(9):827-36.

9.	Moses RG. Gestational Diabetes Mellitus: Implications of an Increased Frequency With IADPSG Criteria. Diabetes Care. 2012;35(3):461-2.

10.	Carolan M. Women's experiences of gestational diabetes self-management: A qualitative study. Midwifery. 2013;29(6):637-45. doi: <https://doi.org/10.1016/j.midw.2012.05.013>.

11.	Wah YYE, McGill M, Wong J, Ross GP, Harding AJ, Krass I. Self-management of gestational diabetes among Chinese migrants: A qualitative study. Women and birth : journal of the Australian College of Midwives. 2019;32(1):e17-e23. Epub 2018/04/26. doi: 10.1016/j.wombi.2018.03.001. PubMed PMID: 29693546.

12.	Kandasamy S, Nguyen L, Desai D, Anand SS, Sherifali D, de Souza RJ. Barriers to, and Facilitators of, Lifestyle Changes to Prevent Gestational Diabetes: An Interpretive Description of South Asian Women and Health-Care Providers Living and Working in Southern Ontario, Canada. Canadian Journal of Diabetes. 2021;45(2):144-54. doi: <https://doi.org/10.1016/j.jcjd.2020.07.001>.

13.	American Diabetes Association A. 14. Management of Diabetes in Pregnancy: Standards of Medical Care in Diabetes—2021. Diabetes Care. 2020;44(Supplement_1):S200-S10. doi: 10.2337/dc21-S014 %J Diabetes Care.

14.	Carolan M, Gill GK, Steele C. Women's experiences of factors that facilitate or inhibit gestational diabetes self-management. BMC Pregnancy Childbirth. 2012;12:99-. doi: 10.1186/1471-2393-12-99. PubMed PMID: 22988897.

15.	Kolivand M, Keramat A, Rahimi M, Motaghi Z, Shariati M, Emamian M. Self-care Education Needs in Gestational Diabetes Tailored to the Iranian Culture: A Qualitative Content Analysis. Iran J Nurs Midwifery Res. 2018;23(3):222-9. doi: 10.4103/ijnmr.IJNMR_108_17. PubMed PMID: 29861762.

16.	Bandura A. Social cognitive theory. The International Encyclopedia of Communication. 1989.

17.	Bandura A. Health promotion by social cognitive means. Health Education & Behavior. 2004;31(2):143-64.

18.	United Nations Relief and Works Agency for Palestine Refugees in the Near East U. World at War (PDF). 2015 3/10/2015. Report No.

19.	United Nations High Commissioner for Refugees U. “Operational Data Report, Jordan, 2022a. 2022a.

20.	Alduraidi H, Saleh, Z., & Hamdan-Mansour, A. Socio-demographic Predictors of Perceived Health among Syrian Refugees in Jordan. Medico Legal Update. 2021;21(1):287-91. doi: 10.37506/mlu.v21i1.2322.

21.	El-Khatib Z, Scales D, Vearey J, Forsberg BC. Syrian refugees, between rocky crisis in Syria and hard inaccessibility to healthcare services in Lebanon and Jordan. Conflict and Health. 2013;7(1):18. doi: 10.1186/1752-1505-7-18.

22.	El Arab R, Sagbakken M. Healthcare services for Syrian refugees in Jordan: a systematic review. European Journal of Public Health. 2018;28(6):1079-87. doi: 10.1093/eurpub/cky103.

23.	Krafft C, Sieverding M, Salemi C, Keo C. Syrian Refugees in Jordan: Demographics, Livelihoods, Education, and Health. 2019. p. 141-72.

24.	Saleh A, Aydin S, Kocak O. A comparative Study of Syrian Refugees in Turkey, Lebanon, and Jordan: Healthcare Access and Delivery. International Journal of Society Systems Science. 2018. doi: 10.26466/opus.376351.

25.	Samari G. Syrian Refugee Women's Health in Lebanon, Turkey, and Jordan and Recommendations for Improved Practice. World Medical and Health Policy. 2017;9:255-74. doi: 10.1002/wmh3.231.

26.	Elliott JA, Das D, Cavailler P, Schneider F, Shah M, Ravaud A, et al. A cross-sectional assessment of diabetes self-management, education and support needs of Syrian refugee patients living with diabetes in Bekaa Valley Lebanon. Conflict and Health. 2018;12(1):40. doi: 10.1186/s13031-018-0174-9.

27.	Ozel S, Yaman S, Kansu-Celik H, Hancerliogullari N, Balci N, Engin-Ustun Y. Obstetric Outcomes among Syrian Refugees: A Comparative Study at a Tertiary Care Maternity Hospital in Turkey. Revista Brasileira de Ginecologia e Obstetrícia. 2018;40.

28.	Turkay Ü, Aydın Ü, Salıcı M, Çalışkan E, Terzi H, Astepe BS, et al. Comparison of pregnant Turkish women and Syrian refugees: Does living as a refugee have an unfavorable effect on pregnancy outcomes? International Journal of Gynecology & Obstetrics. 2020;149(2):160-5. doi: <https://doi.org/10.1002/ijgo.13117>.

29.	Alnuaimi K, Kassab M, Ali R, Mohammad K, Shattnawi K. Pregnancy outcomes among Syrian refugee and Jordanian women: a comparative study. International Nursing Review. 2017;64(4):584-92. doi: <https://doi.org/10.1111/inr.12382>.

30.	Boulle P, Kehlenbrink S, Smith J, Beran D, Jobanputra K. Challenges associated with providing diabetes care in humanitarian settings. The Lancet Diabetes & Endocrinology. 2019;7(8):648-56. doi: <https://doi.org/10.1016/S2213-8587(19)30083-X>.

31.	Adwan MA, Najjar YW. The relationship between demographic variables and diabetes self-management in diabetic patients in Amman city/Jordan. Global journal of health science. 2013;5(2):213.

32.	Al-Khawaldeh OA, Al-Hassan MA, Froelicher ES. Self-efficacy, self-management, and glycemic control in adults with type 2 diabetes mellitus. Journal of Diabetes and its Complications. 2012;26(1):10-6.

33.	Darawad MW, Hammad S, Mosleh S, Samarkandi OA, Hamdan-Mansour A, Khalil AA, et al. Psychosocial correlates of diabetes self-management practices. Iranian Journal of Public Health. 2017;46(6):771.

34.	Albikawi ZF, Abuadas M. Diabetes self-care management behaviours among Jordanian type two diabetes patients. Am Int J Contemp Res. 2015;5(3):87-95.

35.	Al-Amer RM, Sobeh MM, Zayed AA, Al-Domi HA. Depression among adults with diabetes in Jordan: risk factors and relationship to blood sugar control. Journal of Diabetes and its Complications. 2011;25(4):247-52.

36.	(DHS) DaHS. Jordan 2017-18 Population and Family Health Servery (PDF). 2017 6/6/2022. Report No.

37.	solutions s. calculating sample size 2023 [cited 2023 Aprile,30, 2023]. Available from: <https://www.statisticssolutions.com/dissertation-resources/sample-size-calculation-and-sample-size-justification/sample-size-formula/#:~:text=Some%20researchers%20do%2C%20however%2C%20support,minimum%20sample%20size%20of%2030>.

38.	World Health Organization W. A healthy lifestyle - WHO recommendations 6 May 2010 WHO2010 [January, 14, 2023]. Available from: <https://www.who.int/europe/news-room/fact-sheets/item/a-healthy-lifestyle---who-recommendations>.

39.	AlQahtani AH, Alzahrani AS, Alzahrani SH, Alqahtani SM, AlOtaibi AF, Khan AA. Levels of Practice and Determinants of Diabetes Self-Care in Primary Health Care in Jeddah City, Saudi Arabia. Cureus. 2020;12(6):e8816. Epub 2020/08/04. doi: 10.7759/cureus.8816. PubMed PMID: 32742831; PubMed Central PMCID: PMCPMC7384715.

40.	Alkhawaldeh O, Shoqirat N, Abu-sheikh B, Froelicher E. Health-Promoting Lifestyles and Awareness of Diabetes in Adults with Type 2 Diabetes. Jordan medical journal. 2022;in press.

41.	Al Hashmi I, Al Yazidi B, Al Omari O. Translation and psychometric validation of the Arabic Gestational Diabetes Management Self-Efficacy Scale (GDMSES). J Healthc Qual Res. 2022;37(4):231-8. Epub 2022/01/20. doi: 10.1016/j.jhqr.2021.12.004. PubMed PMID: 35042678.

42.	Toobert DJ, Glasgow RE. Assessing diabetes self-management: the summary of diabetes self-care activities questionnaire. Handbook of psychology and diabetes: A guide to psychological measurement in diabetes research and practice. 1994;351:75.

43.	Al-Amer R. Depression and self-care in Jordanian adults with diabetes: The POISE study: University of Western Sydney (Australia); 2015.

44.	Al-Hashmi I, Hodge F, Nandy K, Thomas E, Brecht ML. The Effect of a Self-Efficacy-Enhancing Intervention on Perceived Self-Efficacy and Actual Adherence to Healthy Behaviours Among Women with Gestational Diabetes Mellitus. Sultan Qaboos University medical journal. 2018;18(4):e513-e9. Epub 2019/04/17. doi: 10.18295/squmj.2018.18.04.014. PubMed PMID: 30988972; PubMed Central PMCID: PMCPMC6443280.

45.	Lyles BE, Chua S, Barham Y, Pfieffer-Mundt K, Spiegel P, Burton A, et al. Improving diabetes control for Syrian refugees in Jordan: a longitudinal cohort study comparing the effects of cash transfers and health education interventions. Conflict and Health. 2021;15(1):41. doi: 10.1186/s13031-021-00380-7.

46.	Al-Khawaldeh OA, Al-Hassan MA, Froelicher ES. Self-efficacy, self-management, and glycemic control in adults with type 2 diabetes mellitus. J Diabetes Complications. 2012;26(1):10-6. Epub 2012/01/10. doi: 10.1016/j.jdiacomp.2011.11.002. PubMed PMID: 22226484.

47.	Moghadam SH, Abdolmaleki EY, Alijani F, Afrakoti NB, Ganji J. The Relationship between Social Capital and Self-Efficacy in Women with Gestational Diabetes Mellitus: A Cross Sectional Study. Ethiopian Journal Of Health Sciences. 2020;30(4).

48.	Kim C, McEwen LN, Kieffer EC, Herman WH, Piette JD. Self-efficacy, social support, and associations with physical activity and body mass index among women with histories of gestational diabetes mellitus. The Diabetes Educator. 2008;34(4):719-28.

49.	Bastani F, Zarrabi R. Correlations of Self-Efficacy among Women with Gestational Diabetes. Hayat. 2010;16.

50.	Pinidiyapathirage J, Jayasuriya R, Cheung NW, Schwarzer R. Self-efficacy and planning strategies can improve physical activity levels in women with a recent history of gestational diabetes mellitus. Psychology & health. 2018;33(8):1062-77.

51.	Al-Hashmi I, Hodge F, Nandy K, Thomas E, Brecht M-L. The effect of a self-efficacy-enhancing intervention on perceived self-efficacy and actual adherence to healthy behaviours among women with gestational diabetes mellitus. Sultan Qaboos University medical journal. 2018;18(4):e513.

52.	Mishali M, Omer H, Heymann AD. The importance of measuring self-efficacy in patients with diabetes. Family Practice. 2010;28(1):82-7. doi: 10.1093/fampra/cmq086.

53.	McCleary-Jones V. Health literacy and its association with diabetes knowledge, self-efficacy and disease self-management among African Americans with diabetes mellitus. ABNF Journal. 2011;22(2).

54.	Tharek Z, Ramli AS, Whitford DL, Ismail Z, Mohd Zulkifli M, Ahmad Sharoni SK, et al. Relationship between self-efficacy, self-care behaviour and glycaemic control among patients with type 2 diabetes mellitus in the Malaysian primary care setting. BMC family practice. 2018;19(1):1-10.

55.	Beckerle CM, Lavin MA. Association of self-efficacy and self-care with glycemic control in diabetes. Diabetes Spectrum. 2013;26(3):172-8.

56.	Yang S, Hsue C, Lou Q. Does patient empowerment predict self-care behavior and glycosylated hemoglobin in chinese patients with type 2 diabetes? Diabetes Technol Ther. 2015;17(5):343-8. Epub 2015/02/06. doi: 10.1089/dia.2014.0345. PubMed PMID: 25654752.

57.	Gerçek E, Şen H. Management of Gestational Diabetes Mellitus: Self-efficacy and Perinatal Outcomes. Güncel Pediatri. 2015;13:209-15. doi: 10.4274/jcp.44366.

58.	Rosenstock IM, Strecher VJ, Becker MH. Social learning theory and the Health Belief Model. Health Educ Q. 1988;15(2):175-83. Epub 1988/01/01. doi: 10.1177/109019818801500203. PubMed PMID: 3378902.

59.	Bandura A. Health promotion by social cognitive means. Health Educ Behav. 2004;31(2):143-64. Epub 2004/04/20. doi: 10.1177/1090198104263660. PubMed PMID: 15090118.

60.	Nooshin Peyman, Fatemeh Behzad, Ali Taghipour, Habibollah Esmaily. Assessment of the Effect of a Health Literacy Educational Program for Health Personnel on Promoting Self-Efficacy among Patients with Chronic Diseases. Health System Research. 2016;12(3):350-7.

61.	Saboula NE, Ahmed NA, Rashad RH. Effect of nursing intervention on knowledge, attitude and self-care activities among gestational diabetic women. International Journal of Novel Research in Healthcare and Nursing. 2018;5(2):135-46.
