# Supplementary material for: Factors influencing gestational diabetes self-care among pregnant women in a Syrian Refugee Camp in Jordan
Source: PLoS One. 2024 Feb 15;19(2):e0297051. doi: 10.1371/journal.pone.0297051 (PMC10868830; doi:10.1371/journal.pone.0297051)
Supplement: Attachment — Submitted filename: response to reviewer revise version.docx [file pone.0297051.s004.docx]

**PONE-D-23-20978R1**

**Factors Influencing Gestational Diabetes Self-Care Among Pregnant Women in a Syrian Refugee Camp in Jordan**

**PLOS ONE**

**Response to reviewers**

| Reviewer comments | Response |
| --- | --- |
| **Reviewer #2**  Line 38: Multiple logistic regression | Well considered, thank you . |
| Line 216: ... keep in mind that this scale) - hanging sentence | Thank you for the review. This sentence was deleted. |
| Line 242: Multiple linear regression *please correct whether linear or logistic | Thank you , this was changed to multiple logistic regression |
| Table 1: characteristics - Knowledgee on diabetes? or general health (please correct) | Thank you , this was Corrected to Knowledge on diabetes |
| Line 279 - typo Gestational Diabetes ..... (please correct) | Corrected , thank you |
| Line 303: R superscript 2 | changed |
| I propose considering professional proofreading services or engaging in peer reviews to thoroughly review the manuscript. | Well considered, Proof reading was done. |
| **Reviewer #3**  Thank you for addressing all the comments well. The article looks much better now with clear objectives and learning points. | Thank you for your valuable time to review our work, we highly appreciate that. |
